# Spinal Stroke: Outcome Attenuation by Erythropoietin and Carbamylated Erythropoietin and Its Prediction by Sphingosine-1-Phosphate Serum Levels in Mice

**DOI:** 10.3390/ijms23179558

**Published:** 2022-08-23

**Authors:** Leon-Gordian Koepke, Edzard Schwedhelm, Wiebke Ibing, Alexander Oberhuber, Guenter Daum, Brigitta Vcelar, Hubert Schelzig, Florian Simon

**Affiliations:** 1 Department of Trauma Surgery and Orthopedic Surgery, University Medical Center Hamburg-Eppendorf, 20251 Hamburg, Germany; 2 Institute for Clinical Pharmacology and Toxicology, University Medical Center Hamburg-Eppendorf, 20251 Hamburg, Germany; 3 German Center for Cardiovascular Research (DZHK e.V.), Partner Site Hamburg/Kiel/Lübeck, 20251 Hamburg, Germany; 4 Clinic for Vascular and Endovascular Surgery, University Hospital Duesseldorf, Heinrich-Heine-University Duesseldorf, 40225 Düsseldorf, Germany; 5 Clinic for Vascular and Endovascular Surgery, University Hospital Münster, 48149 Münster, Germany; 6 Department of Vascular Medicine, University Heart and Vascular Center Hamburg, 20251 Hamburg, Germany; 7 Polymun Scientific Immunbiologische Forschung GmbH, 3400 Klosterneuburg, Austria

**Keywords:** stroke, spinal cord ischemia, spinal cord injury, erythropoietin, endoplasmic reticulum, unfolded protein response, sphingosine-1-phosphate, biomarker

## Abstract

Spinal strokes may be associated with tremendous spinal cord injury. Erythropoietin (EPO) improves the neurological outcome of animals after spinal cord ischemia (SCI) and its effects on ischemia-induced endoplasmic reticulum (ER) stress and the unfolded protein response (UPR) are considered possible molecular mechanisms. Furthermore, sphingosin-1-phosphate (S1P) is suggested to correlate with SCI. In this study, the effect of recombinant human EPO (rhEPO) and carbamylated EPO (cEPO-Fc) on the outcome of mice after SCI and a prognostic value of S1P were investigated. SCI was induced in 12-month-old male mice by thoracic aortal cross-clamping after administration of rhEPO, cEPO-Fc, or a control. The locomotory behavior of mice was evaluated by the Basso mouse scale and S1P serum levels were measured by liquid chromatography-tandem mass spectrometry. The spinal cord was examined histologically and the expressions of key UPR proteins (ATF6, PERK, and IRE1a, caspase-12) were analyzed utilizing immunohistochemistry and real-time quantitative polymerase chain reaction. RhEPO and cEPO-Fc significantly improved outcomes after SCI. The expression of caspase-12 significantly increased in the control group within the first 24 h of reperfusion. Animals with better locomotory behavior had significantly higher serum levels of S1P. Our data indicate that rhEPO and cEPO-Fc have protective effects on the clinical outcome and neuronal tissue of mice after SCI and that the ER is involved in the molecular mechanisms. Moreover, serum S1P may predict the severity of impairment after SCI.

## 1. Introduction

The anterior spinal artery syndrome, as well as surgical procedures on the aorta, are common causes of spinal strokes, potentially resulting in irreversible paraplegia. Many surgical and pharmacological strategies have been tested to protect the spinal cord during procedures on the aorta [1,2,3,4]; however, no generally accepted regimen was established.

Over the last years, the use of erythropoietin (EPO) and its carbamylated derivates (cEPO) have slipped into focus. It was shown in animal models, that EPO and cEPO may attenuate damage after spinal cord ischemia (SCI) [5,6,7]. However, it is unclear how EPO or cEPO might mediate their protective effects during spinal ischemia and reperfusion sequence (IRS).

The endoplasmic reticulum (ER) is crucial in the neuronal response to IRS via the unfolded protein response (UPR) [8]. A key role herein is played by the 78-kDa glucose-regulated protein (GRP78) maintaining the ER homeostasis [9]. GRP78 binds to the ER transmembrane proteins activating transcription Factor 6 (ATF6), protein kinase RNA-like endoplasmic reticulum kinase (PERK), and inositol-requiring enzyme 1α (IRE1α) [10]. Upon accumulation of misfolded protein within the ER, the UPR is initiated by dissociation of ATF6, IRE1α, and PERK from GRP78 [8,11] inducing different pathways, such as the activation of caspase-12 [12].

Furthermore, sphingosine-1-phosphate (S1P) was the focus of research on the neurological outcomes after ischemic stroke and SCI [13,14,15]. It was reported that S1P is elevated at the site of SCI in rats and enhances the plasticity of neuronal progenitor cells [16]. Additionally, the S1P receptor 1 (S1PR1) agonist FTY720 promotes spinal cord recovery after injury [17,18].

This study now investigates the hypothesis of whether EPO or cEPO attenuate the neuronal damage that is caused by spinal IRS in mice via modulation of the UPR and whether serum levels of S1P correlate with neurological function after SCI.

## 2. Results

### 2.1. Neurological Outcome

The results of the Basso mouse scale (BMS) are shown in Table 1. Among the animals with a total reperfusion time of 96 h significantly lower values of BMS were found in the control group (CG) compared to rhEPO and cEPO-Fc at all time points (*p* < 0.05) (Table 1).

### 2.2. Histological and Immunohistochemical Tissue Analysis

**Figure 1:**
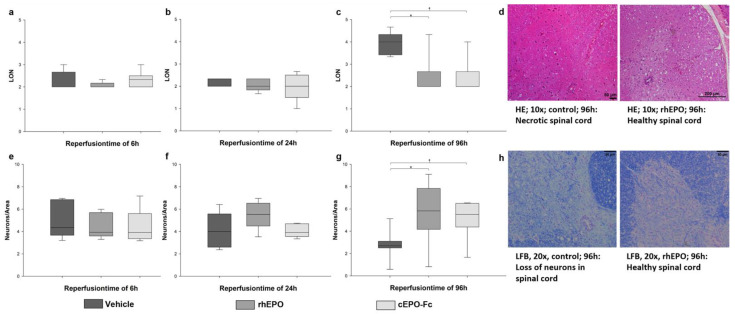
The results of the histopathology. The control group is in dark grey, the rhEPO group is in semi-dark grey, and the cEPO-Fc group is in light grey. Boxes indicate the upper and lower quartile, the line indicates the median, and the upper and lower whiskers indicate 2.5%- and 97.5% quantile. (**a**): Results of the scoring of the level of necrosis (LON) in the hematoxylin eosin (HE) staining after 6 h (h) of reperfusion time (RT). (**b**): LON in HE after 24 h RT. (**c**): LON in HE after 96 h RT. * *p* < 0.05 (Multiple group comparison via Kruskal–Wallis test and post hoc analysis using Dunn’s test). (**d**): HE in 10×. Necrotic spinal cord in the control group and healthy spinal cord in the rhEPO group after 96 h RT. (**e**): Results of the counting of the neurons/area (N/A) in the ventral horn of the spinal cord after 6 h RT. (**f**): N/A in the ventral horn of the spinal cord after 24 h RT. (**g**): N/A in the ventral horn of the spinal cord after 96 h RT * *p* < 0.05 (Multiple group comparison via Brown–Forsythe test and post hoc analysis using Bonferroni test). (**h**): LFB in 20×. Loss of neurons in the control group healthy spinal cord in rhEPO group after 96 h RT.

The results of the hematoxylin-eosin (HE) and Luxol Fast Blue (LFB) stainings are shown in Figure 1a–h. In the HE staining, significantly higher levels of necrosis (LON) were obtained in the CG compared to rhEPO and cEPO-Fc, at 96 h (*p* < 0.001) (Figure 1c). The LFB staining showed significantly fewer neurons per area in the CG when compared to rhEPO (*p* < 0.001) or cEPO-Fc (*p* = 0.01), at 96 h (Figure 1g).

**Figure 2:**
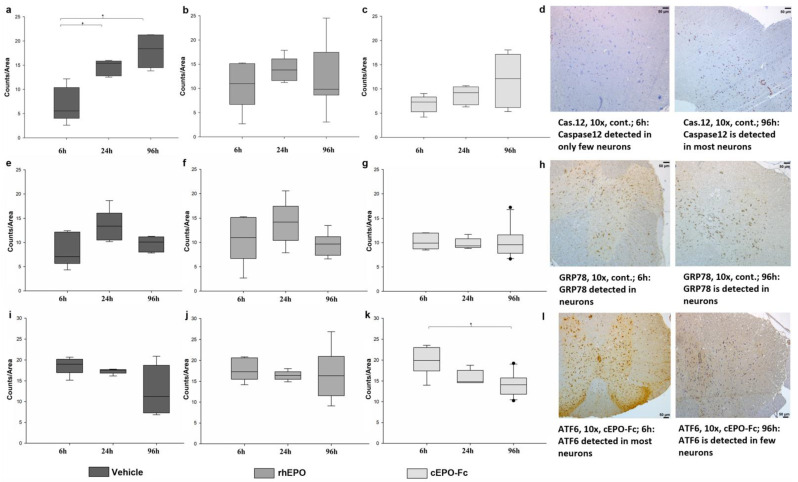
The results of the immunohistochemistry. The control group is in dark grey, the rhEPO group in semi-dark grey, and the bcEPO-Fc group is in light grey. Boxes indicate the upper and lower quartile, and the line indicates the median, and the upper and lower whisker indicate 2.5%- and 97.5% quantile, and black circles indicate values that are outside 2.5%- and 97.5% quantile. (**a**): Results of the immunohistochemical (IHC) evaluation of caspase-12 in the control group after 6 h, 24 h, and 96 h RT. * 6 h vs. 24 h *p* = 0.005 and 6 h vs. 96 h *p* < 0.001 (Multiple group comparison via Brown–Forsythe test and post hoc analysis using Bonferroni test) (**b**): IHC of caspase-12 in the rhEPO group after 6 h, 24 h, and 96 h RT. (**c**): IHC of caspase-12 in the cEPO-Fc group after 6 h, 24 h, and 96 h RT. (**d**): IHC of caspase-12 in 10×. Caspase-12 is detected in only few neurons after 6 h RT in the control group. Caspase-12 is detected in most neurons after 96 h RT in the control group. (**e**) IHC of GRP78 in the control group after 6 h, 24 h, and 96 h RT. (**f**): IHC of GRP78 in the rhEPO group after 6 h, 24 h, and 96 h RT. (**g**): IHC of GRP78 in the cEPO-Fc group after 6 h, 24 h, and 96 h RT. (**h**): IHC of GRP78 in 10×. GRP78 is detected in neurons after 6 h and 96 h RT in the control group. (**i**): IHC of ATF6 in the control group after 6 h, 24 h, and 96 h RT. (**j**): IHC of ATF6 in the rhEPO group after 6 h, 24 h, and 96 h RT. (**k**): IHC of ATF6 in the cEPO-Fc group after 6 h, 24 h, and 96 h RT. * 6 h vs. 96 h *p* = 0.003 (Multiple group comparison via Brown–Forsythe test and post hoc analysis using Bonferroni test). (**l**): IHC of ATF6 in 10×. GRP78 is detected in neurons after 6 h and 96 h RT in the control group.

The results of the immunohistochemistry are shown in Figure 2a–l. In the CG, significantly less caspase-12 was detected at 6 h than at 24 h (*p* = 0.004) or 96 h (*p* = 0.005) (Figure 2a). In contrast, in the rhEPO (Figure 2b) and cEPO-Fc groups (Figure 2c) no time-dependent changes in caspase-12 expression were found. In the cEPO-Fc group, ATF6 expression is significantly higher after 6 h than after 96 h (*p* = 0.003) (Figure 2k). No further significant differences were detected for GRP78 or ATF6 between the groups or reperfusion time periods (Figure 2e–l).

**Figure 3:**
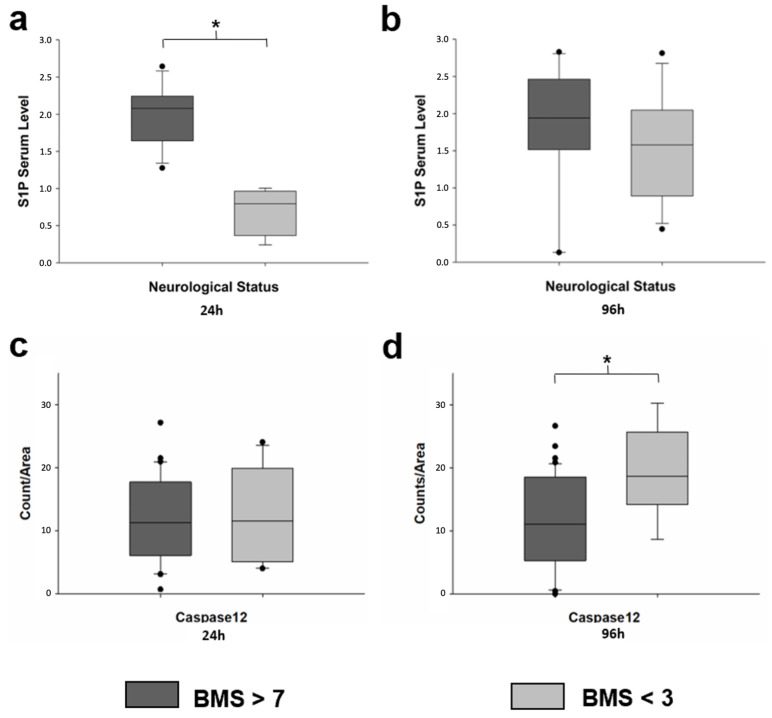
Serum level of S1P and immunohistochemistry for caspase-12 of animals with a BMS > 7 and < 3. Basso mouse scale (BMS) > 7 in dark grey and BMS < 3 in light grey. Boxes indicate the upper and lower quartile, the line indicates the median, the upper and lower whisker indicate 2.5%- and 97.5% quantile, and black circles indicate values that are outside 2.5%- and 97.5% quantile. (**a**): Serum levels of S1P of after 24 h RT. * BMS > 7 (*n* = 11) vs. <3 (*n* = 4) *p* < 0.001 (*t*-test). (**b**): Serum levels of S1P after 96 h. (**c**): Results of the immunohistochemical (IHC) evaluation of caspase-12 after 24 h. (**d**): IHC of caspase-12 after 96 h. * BMS > 7 (*n* = 18) vs. <3 (*n* = 16) *p* = 0.009 (*t*-test).

At 96 h, regardless of group affiliation, animals with a BMS > 7 show significantly lower caspase-12 levels than animals with a BMS < 3, (*p* = 0.009) (Figure 3d).

### 2.3. Serum Levels of Sphingosine-1-Phosphate

Animals with a BMS > 7 showed significantly higher S1P serum levels after 24 h than animals with a BMS < 3 (Figure 3a) (*p* < 0.001). This difference was no longer present after 96 h, as S1P serum levels increased in animals with a BMS < 3 (Figure 3b).

### 2.4. Gene Expression

The results of the analysis of IRE1α, PERK, and ATF6 by RT-qPCR are shown in Table 2. Within the c-EPO-Fc group, expression of IRE1α (*p* = 0.028) as well as of PERK (*p* = 0.035) at 6 h was significantly lower than at 24 h (Table 2). Furthermore, after 24 h, there were significantly higher expressions of IRE1α in the cEPO-Fc group than in the rhEPO group (*p* = 0.033), and significantly higher expressions of PERK in the cEPO-Fc group than in the rhEPO group (*p* = 0.012) and the CG (*p* = 0.032).

## 3. Discussion

The major findings of this study are (1) presurgical treatment with rhEPO and cEPO-Fc both significantly improve the neurological outcome of mice; (2) compared to the controls, a significantly higher number of intact neurons was found in both EPO treatment groups after 96 h; (3) the expression of the ER-specific proapoptotic caspase-12 significantly increases within the CG during the first 24 h of reperfusion; and (4) regardless of treatment, there is an association between BMS and serum-S1P concentrations.

Recently, it was shown that rhEPO and cEPO-Fc have beneficial effects on SCI in large and small animal models [5,7,19]. In this study, preoperative conditioning using rhEPO and cEPO-Fc was shown to be associated with a significantly improved neurological outcome of mice after SCI compared with CG. Furthermore, histopathology was used to quantify the effects of the treatment with rhEPO and cEPO-Fc. By HE and LFB staining, necrosis, and loss of neurons in the spinal cord were detected after more than 24 h. As described before, destruction of motoneurons after SCI takes place from 2 to 7 days (d) of reperfusion [20,21]. In this study, the animals that were treated with rhEPO and cEPO-Fc showed significantly less tissue damage in the spinal cord after 96 h, confirming previous observations [7,20,22].

The loss of neurons in ischemic damaged neuronal tissue might indicate involvement of the proapoptotic caspase-12; caspase-12 is specifically activated by ER-stress (ERS) [23,24,25]. After activation, caspase-12 is capable of activating other Caspases, (e.g., Caspase 3 and 9), triggering a chain reaction finally resulting in apoptosis [26]. The expression of caspase-12 correlates with the extent of damage of the spinal cord after an injury [21] and inhibition of caspase-12 protects the spinal cord [27] and improves neurological recovery [28]. In this study, the expression of caspase-12 in the tissue of the CG increases significantly between 6 and 24 h. Previously, it was described that caspase-12 is strongly detectable after 8 h after SCI but already decreased after 1 d of reperfusion [21,29].

The rise of expression of caspase-12 in the CG corresponds with the results of the HE and LFB staining, as it goes ahead in time of the formation of necrosis and a loss of neurons. The higher expression of caspase-12 in the CG is associated with a lower number of intact neurons and impaired neuronal function. Furthermore, regardless of the group affiliation, animals with a good neurological outcome (BMS > 7) showed significantly less caspase-12 than animals with a poor neurological outcome (BMS < 3), at 96 h. From the current findings, it can be concluded that high expression of caspase-12 correlates with the formation of necrosis in the spinal cord and an impaired neurological outcome.

The induction of GRP78 expression in ERS and a positive correlation between the expression of GRP78 and caspase-12 has previously been described [23,25,29]. However, although GRP78 expression was detectable in the CG and treatment groups after 6, 24, and 96 h, the expression did not show significant differences between the groups. Although it was described that intravenous administration of sodium 4-phenylbutyrate in rabbits during spinal IRS prevented the expression of GRP78 in the spinal cord of the animals [29], these effects could not be demonstrated for rhEPO and cEPO-Fc in this study.

Upon the onset of ERS, GRP78 dissociates from ATF6, PERK, and IRE1α. Subsequently, these three key proteins can initiate different pro- or anti-apoptotic cascades and the UPR is activated [8,11]. However, after 6 h, no significant differences in the expression of any of the three proteins were detected between the three study groups. After 24 h, the cEPO-Fc group showed significantly higher expression of IRE1α when compared to the rhEPO group and significantly higher expression of PERK when compared to the rhEPO group and CG, respectively. An elevation of IRE1α expression at 1 day after traumatic spinal cord injury in rats [30] and the potential of IRE1α activating caspase-12 were described before [24]. Therefore, we expected the increased expression of IRE1α after 24 h in the cEPO-Fc group to be associated with an increased expression of caspase-12 in the tissue. However, after 24 h, a significantly lower expression of caspase-12 was detected in the cEPO-Fc group compared to the other groups. It can be hypothesized that an elevation of IRE1α does not necessarily lead to an increased expression of caspase-12. Since PERK is thought to have mainly proapoptotic effects in the context of the UPR [10], we expected to find lower expression of PERK in animals of the EPO treatment groups. This, however, was not the case. But, before, it was also reported that the PERK branch of the UPR promotes neuronal function via the suppression of translation after stroke in mice [31]. This supports the current findings, as elevated levels of PERK do not necessarily lead to increased damage of tissue and impaired neurologic function. The analysis of ATF6 gene expression did not yield any significant findings between the study groups. Recently, it was shown in adult zebrafish that ATF6 levels increase at 12 h after traumatic spinal cord injury and it was suggested that ATF6 improves neurological function [32]. In general, the expression of ATF6 might be important in SCI as it is preserved in many species. Overall, the exact processes that are involved in mediating the beneficial effects of rhEPO and cEPO-Fc remain to be fully elucidated. However, the influence of ERS appears to be paramount and further studies in animal models should be performed in this regard.

S1P is elevated in the central nervous system at the injured site, and it might work as a chemoattractant factor that is involved in the migration of neural stem and progenitor cells [16]. Moreover it was shown in rodent models that (FTY-720) can promote recovery after SCI due its immunosuppressive effects [18,33] and that modulation of S1P receptors has beneficial effects on neuronal survival [34]. The data from this study show that after 24 h the blood levels of S1P were significantly higher in animals with a good neurological outcome, regardless of group affiliation. The current findings suggest that high blood levels of S1P after 24 h correlate with an improved neurological outcome after SCI, supporting previous findings [17,33,34]. However, higher levels of S1P after 96 h were seen in all animals regardless of outcome. It is possible that high levels in the early phase after SCI are crucial for the subsequent neurological outcome. This finding is in line with previous reports in clinical and experimental ischemic stroke [13,14]. In mice that were subjected to middle cerebral artery occlusion, immediate activation of endothelial S1PR1 prevents endothelial leakage and reduces ischemic damage and neuronal death [14]. These effects were limited to the first hours after vascular damage and were independent of lymphopenia that is induced by chronic activation of lymphocyte S1PR1 over several days.

Today, it is still difficult to prognose the outcome after SCI and potential biomarkers are in the current focus of research [35]. As S1P can be determined in blood samples and as shown by the current data, early high S1P serum levels after SCI correlate with good neurological outcomes, S1P might be useful as a prognostic marker after SCI. Studies in animal models and especially in humans must follow to further investigate the potential of S1P as a biomarker to predict clinical outcomes after damage to the spinal cord.

## 4. Materials and Methods

### 4.1. Animal Model

All animal procedures were approved by the University Animal Care Committee and the Federal Authorities for animal research (file number: 84-0204.201.A081). Experiments were performed with 12-month-old male C57BL/6 mice (Janvier Labs, Le Genest-Saint-Ilse, Frace). Preoperatively, 5000 I.U./kg of recombinant human erythropoietin (rhEPO) (Roche, Basel, Switzerland), 50 µg/kg of carbamylated erythropoietin-Fc fusion protein (cEPO-Fc) (Polymun Scientific Immunbiologische Forschung GmbH, Klosterneuburg, Austria) [36], or control vehicle (phosphate buffered saline (PBS) were once administered intraperitoneally (i.p.), after randomization by lot. A left-sided thoracotomy was performed, and the descending thoracic aorta was dissected. The descending thoracic aorta distally of the left subclavian artery and the left subclavian artery itself were then clamped for 7 min. Postoperatively, the animals were followed up for the reperfusion periods of 6, 24, and 96 h until sacrifice by heart puncture, in deep anesthesia. The animals were evaluated every 12 h by the Basso mouse scale (BMS) [37]. The BMS is an observational score in which locomotion defects in mice are assessed with 0–9 points. Roughly, the mobility of the hind limbs as well as the tail and trunk stability are assessed. 0 points correspond to a complete loss of function in the evaluation, while 9 points indicate normal function. Animals that were examined for a total reperfusion period of 6 h were neurologically evaluated before sacrifice. Blood samples were collected by puncture of the left ventricle with a 1 mL syringe (B-braun, Melsungen, Germany) and a 22 G needle (B-braun, Melsungen, Germany). Whole blood was immediately transferred to a 1.5 mL Eppendorf tube (Eppendorf, Wesseling, Germany) and allowed to clot at room temperature for 1 h. The blood was then centrifuged at 1000× *g* for 10 min at 4 °C. The supernatant was pipetted into a fresh 1.5 mL Eppendorf tube (Eppendorf, Wesseling, Germany) and stored at −80 °C until measurement. After sacrifice, the spinal cord was excised and divided into three sections and each section was again divided in half. One half was fixed in 4% buffered formalin solution and embedded into paraffin, the other one was snap-frozen in liquid nitrogen and cryopreserved at −80 °C. The study groups and animal numbers that were used are summarized in Table 3.

### 4.2. Histology

The formalin-fixed specimens were stained with HE and LFB to assess the general morphology and number of neurons, respectively. Immunohistochemistry (IHC) was performed for caspase-12 (1:500; Gene ID: 12364; Sigma-Aldrich, St. Louis, MO, USA), GRP78 (1:250; Gene ID: 3309; Novus Biologicals, Littleton, CO, USA) and ATF6 (1:300; Clone: 70B1413.1; Novus Biologicals, Littleton, CO, USA). The IHC stainings were performed as previously described techniques [38]. In summary, for the preparation of the IHC stains, two paraffin sections were placed on each adhesive slide: one for labeling with the respective antibody, and the other served as a negative control. At the beginning of staining, the sections were deparaffinized using a Roticlear (Carl Roth, Karslruhe, Germany) and the water was removed in an alcohol series. This was followed by antigen unmasking by boiling the sections for 10 min at 100 °C in citrate buffer (pH 6.0) and subsequent incubation with the respective antibody (caspase-12, GRP78 or ATF6) and the negative control (rabbit immunoglobulin G (IgG) for caspase-12 and GRP78 and mouse IgG for ATF6). For caspase-12, antigen unmasking could be omitted. Blocking was performed using horse serum (caspase-12) or bovine serum albumin (GRP78 and ATF6). After incubation of the antibodies, incubation was performed using a biotinylated antibody (biotinylated rabbit IgG for caspase-12 and GRP78 and biotinylated mouse IgG for ATF6) (Vector Laboratories Inc., Burlingame, CA, USA) followed by streptavidin horseradish proteases (Dako, Jena, Germany). Next, color was developed using an AEC kit (caspase-12) (Sigma-Aldrich, St. Louis, MO, USA) or a DAB kit (GRP78 and ATF6). (Merck, Darmstadt, Germany). Finally, counterstaining was performed using hematoxylin according to Gill II (Carl Roth, Karslruhe, Germany).

### 4.3. Histological Evaluation

**Figure 4:**
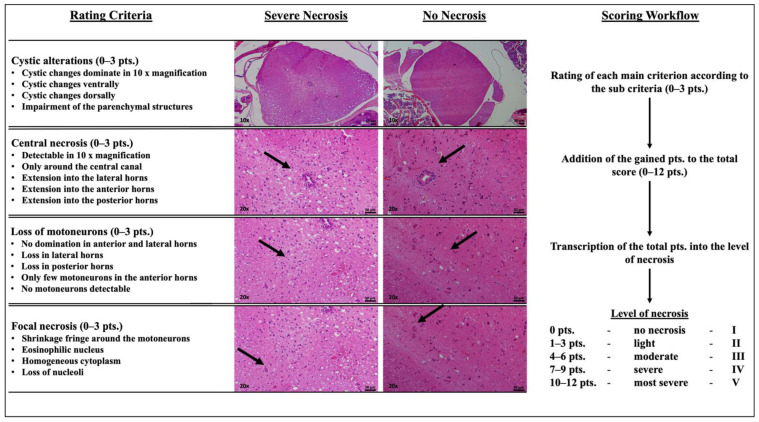
Workflow of the hematoxylin eosin necrosis score. This figure shows the workflow for evaluating the extent of necrosis in murine spinal cord based on hematoxylin eosin staining using a semiquantitative scoring system. There were four main criteria that were defined for the evaluation of necrosis. The arrows indicate the respective region or structure of interest for the evaluation of the main criterion. Each of these criteria was evaluated semi-quantitatively, according to sub-criteria, with 0–3 points (pts.). This results in a possible total score of 0–12 pts. This total score is translated into levels of necrosis (LON) from I–V as shown in the figure.

Using the HE-stained tissue sections, a necrosis score was developed and assessed to quantify the level of tissue necrosis form I-V (LON) (Figure 4).

In LFB-stained tissue sections, the number of motoneurons that were located in the ventral horn in relation to its surface was determined. The evaluation of immunohistochemical staining was performed with software that was supported with a toolbox for Fiji software (Version 2.5.6, BioVoxxel, Mutterstadt, Germany), by counting the number of detections in the ventral horn in relation to its surface [38].

### 4.4. Gene Expression

The analysis of expression of IRE1α, PERK, and ATF6 was performed by real-time quantitative polymerase chain reaction (RT-qPCR). The spinal cord was dissected from the deep-frozen tissue for RNA extraction. It was homogenized in TRIzol (Ambion by Life Technologies, Carlsbad, CA, USA) followed by the precipitation of RNA by incubation in 100% isopropanol, centrifugation, and washing of the RNA pellet in first 100% and then 75% ethanol. The synthesis of cDNA was then performed using the High-Capacity cDNA Reverse Transcription Kit from Applied Biosystems^TM^ (Thermo Fisher Scientific, Waltham, MA, USA). RT-qPCR was performed using ribosomal RNA 18S (18SrRNA) as a reference gene. The primers that were used were purchased from Qiagen (Qiagen N.V., Venlo, The Netherlands). The purchasing details are stated in Table 4. Data analysis and relative quantification was performed by using the ddCt method, as described before [39].

### 4.5. Measurement of Serum Sphingosine-1-Phosphate

Serum was obtained from a puncture of the left ventricle with subsequent centrifugation and storage at −80 °C until analysis. S1P was measured by liquid chromatography-tandem mass spectrometry (LC-MS/MS) as previously described [40]. In brief, 20 µL of serum were added to 20 µL of 1 µmol/L stable isotope-labelled internal standard d_7_-S1P (Avanti Polar Lipids, Alabaster, AL, USA) solved in methanol and 80 µL of acetonitrile/water, 80/20, vol/vol. Supernatants were cleared by centrifugation and subjected to ultra-performance liquid chromatography on an AQUITY UPLC BEH C8 1.7 µm column (2.1 × 75 mm, Waters, Eschborn, Germany) using an elution gradient of the two mobile phases (A) 0.1% formic acid in water and (B) 0.1% formic acid in acetonitril, at a flow rate of 0.4 mL/min over 4.2 min. Quantification was performed on a Xevo Triple Quadrupole Mass Spectrometer (Waters) with positive electrospray ionization in the multiple reaction mode. Peak area ratios of analyte and internal standard were calculated for calibration (four levels) and quality control (QC-low and -high) of the samples and used for quantification. If coefficients of variation for QCs were above 10%, samples were re-analyzed.

### 4.6. Statistical Analysis

Statistical analyses were performed using SigmaPlot 13 software (Systat Software Inc., San Jose, CA, USA). Randomization of animals was by lot. Testing for normal distribution was performed using Kolmogorov–Smirnov tests. In the case of comparison of two groups, normally (non-normally) distributed datasets were analyzed by *t*-test (Mann–Whitney-Rank sum test). For multiple group comparisons, statistical testing was done by Brown-Forsythe (Kruskal–Wallis) tests with post hoc analyses using the Bonferroni or Tukey (Dunn’s) test. The significance level was *p* < 0.05.

## 5. Conclusions

It was shown that both rhEPO and cEPO-Fc have beneficial effects on the clinical outcome of mice after spinal IRS and attenuate damage to the spinal cord. Furthermore, it was shown that the expression of caspase-12 is elevated with prolonged reperfusion time in the CG. Even though the clinical and histological effects of rhEPO and cEPO-Fc were protective against SCI and the ER seems to be involved in this process, the underlying pathway stayed unclear. This topic should be the aim of further investigations. Next to that, it was shown that high S1P serum levels early after SCI are associated with good prognosis in mice and could thus be useful as a marker for the prognosis of patients, too. Further investigation in patients might also identify individuals that benefit from the activation of S1PR1 by FTY-720 early after ischemic damage.

## Figures and Tables

**Table 1:** Evaluation of the neurological status using the Basso-mouse-scale.

Reperfusion Group (h)	Reperfusion Time (h)	BMS Control	BMS rhEPO	BMS cEPO-Fc
6	6	2.8 ± 2.4	1.8 ± 1.2	0.5 ± 1.1
24	12	5.6 ± 4.2	8.5 ± 0.6	5.4 ± 4.4
24	24	5.6 ± 4.2	8.6 ± 0.5	5.4 ± 4.4
96	12	1.3 ± 2.8	7.1 ± 3.5 *	7.1 ± 2.7 ^§^
96	24	1.1 ± 2.4	7.1 ± 3.4 *	6.9 ± 3.0 ^§^
96	36	1.2 ± 2.4	7.5 ± 3.2 *	7.2 ± 3.1 ^§^
96	48	1.3 ± 2.4	7.0 ± 3.7 *	7.3 ± 3.1 ^§^
96	60	1.2 ± 2.4	7.3 ± 3.6 *	7.2 ± 3.1 ^§^
96	72	1.3 ± 2.4	7.3 ± 3.6 *	7.2 ± 3.1 ^§^
96	84	1.2 ± 2.4	7.3 ± 3.6 *	7.3 ± 3.1 ^§^
96	96	1.3 ± 2.4	7.3 ± 3.6 *	7.4 ± 3.2 ^§^

Data are listed from left to right according to reperfusion group (6, 24, and 96 h), measurement after reperfusion time (6–96 h) and treatment group (control, rhEPO, cEPO-Fc). The data shown in means and standard deviation. BMS = Basso mouse scale; cEPO-Fc = carbamylated erythropoietin FC fusion protein; h = hours; rhEPO = recombinant human erythropoietin. * rhEPO vs. control—*p* < 0.05. ^§^ cEPO-Fc vs. control—*p* < 0.05 (Multiple group comparison via Kruskal–Wallis test and post hoc analysis using Dunn’s test).

**Table 2:** RT-qPCR measurements of ATF6, IRE1α, and PERK in the spinal cord of the animals of the control, recombinant human erythropoietin (rhEPO), and carbamylated erythropoietin FC fusion protein (cEPO-Fc) group after 6 and 24 h of reperfusion.

Detected Gene	Reperfusion Time (h)	Control	rhEPO	cEPO-Fc
ATF6	6	1.16 ± 0.75	1.11 ± 0.32	1.29 ± 0.99
	24	1.20 ± 0.61	2.20 ± 1.77	1.48 ± 1.16
IRE1α	6	1.06 ± 0.45	0.95 ± 0.21	0.93 ± 0.29 *
	24	1.10 ± 0.47	0.80 ± 0.21	2.35 ± 1.31 ^&^
PERK	6	1.13 ± 0.64	1.08 ± 0.28	1.04 ± 0.61 *
	24	1.05 ± 0.36	0.80 ± 0.08	2.40 ± 1.17 ^§^

Data are shown in means and standard deviation (Fold change). cEPO-Fc = carbamylated erythropoietin FC fusion protein; h = hours; rhEPO = recombinant human erythropoietin. * Within one group 6 h vs. 24 h—*p* < 0.05 (two-tailed t-test). ^&^ cEPO-Fc vs. rhEPO at 24 h—*p* = 0.033 (Multiple group comparison via Brown–Forsythe test and post hoc analysis using Tukey test). ^§^ cEPO-Fc vs. rhEPO and control at 24 h—*p* = 0.012 (cEPO-Fc vs. rhEPO and *p* = 0.032 (cEPO-Fc vs. control) (Multiple group comparison via Brown–Forsythe test and post-hoc analysis using Tukey test).

**Table 3:** Study groups and the number of animals.

Reperfusion Time (h)	Study Group	Number of Animals (*n*)
6	control	5
6	rhEPO	5
6	cEPO-Fc	6
24	control	5
24	rhEPO	5
24	cEPO-Fc	5
96	control	13
96	rhEPO	10
96	cEPO-Fc	11

h = hours; *n* = number of animals; cEPO-Fc = carbamylated erythropoietin FC fusion protein; rhEPO = recombinant human erythropoietin.

**Table 4:** Primer purchasing detail.

Gene of Interest	Order Number at Qiagen N.V.
ATF6	PPM33057A
IRE1α (Em1)	PPM36937A
PERK (Eif2ak3)	PPM26428B
18SrRNA	PPM57735E

ATF6 = activating transcription factor 6, IRE1α = inositol-requiring enzyme 1α, PERK = protein kinase RNA-like endoplasmic reticulum kinase, 18S ribosomal RNA.

## Data Availability

The data that were generated or analyzed during this study are included in this published article. A summary of the data can be provided upon request.
